# Ferulic Acid Alleviates Chemotherapy-Induced POI by Targeting the Grp78 and Perk-eIF2α-ATF4-CHOP Pathway to Attenuate Endoplasmic Reticulum Stress

**DOI:** 10.3390/biomedicines14030714

**Published:** 2026-03-19

**Authors:** Fan Li, Yanjing Huang, Zhuo Liu, Yuli Geng, Runan Hu, Yufan Song, Lijun Xu, Mingmin Zhang

**Affiliations:** 1 Institute of Integrated Traditional Chinese and Western Medicine, Tongji Medical College, Huazhong University of Science and Technology, Wuhan 430030, China; fern5933@163.com (F.L.);; 2 Tongji Medical College, Huazhong University of Science and Technology, Wuhan 430030, China

**Keywords:** ferulic acid, premature ovarian insufficiency, cyclophosphamide endoplasmic reticulum stress, Perk/eIF2α/ATF4/CHOP, apoptosis

## Abstract

**Backgrounds**: Premature ovarian insufficiency (POI) is a clinical syndrome characterized by premature ovarian dysfunction, amenorrhea, and infertility. Ferulic acid (FA) is a prominent bioactive phenolic compound derived from traditional Chinese herbs *Angelica sinensis* (Oliv.) Diels and *Ligusticum chuanxiong* Hort. These herbs are commonly used to treat gynecological disorders including menstrual irregularities and infertility, and are known to modulate endoplasmic reticulum (ER) stress. However, the therapeutic potential and molecular mechanisms of FA in the context of POI remain largely unexplored. This study aimed to investigate the protective effects of FA against POI and to elucidate the underlying pharmacological mechanisms. **Methods**: In vivo, a mouse model of POI was established via a single intraperitoneal injection of cyclophosphamide (CTX; 120 mg/kg), and using FA for 28 days of continuous gavage to observe its therapeutic effect. Ovarian function and pathological changes were assessed by hormone levels, follicle development and oxidative stress (OS) level. In vitro, the effects of FA were examined using 4-hydroperoxy cyclophosphamide (4-OHCP)-treated KGN granulosa cells. Transcriptome sequencing, molecular docking, and molecular dynamics simulations were employed to identify potential targets of FA. **Results**: Our findings demonstrated that FA administration helped preserve regular estrous cycles, promoted follicle development and hormone secretion, and attenuated OS in both ovarian tissue and granulosa cells (GCs). Transcriptomic profiling combined with molecular docking and molecular dynamics simulations suggested that FA potentially targets key ER stress proteins, specifically Grp78 and Perk. Further in vivo and in vitro experiments confirmed that FA alleviates ER stress by inhibiting the overactivation of the Perk/eIF2α/ATF4/CHOP signaling pathway. Notably, the protective effects of FA were comparable to those of the ER stress inhibitor 4-Phenylbutyric acid (4-PBA) and were reversed by the ER stress activator tunicamycin (TM). Additionally, FA downregulates ERO1α expression, further blocking secondary oxidative damage triggered by ER stress. In KGN cells, FA significantly inhibits 4-OHCP-induced apoptosis and upregulates the anti-apoptotic proteins BCL-2 and BCL-xL, exhibiting efficacy similar to 4-PBA. **Conclusions**: FA improves ovarian function in CTX-induced POI by coordinately regulating OS and ER stress, inhibiting the Perk/eIF2α/ATF4/CHOP pathway, and suppressing GC apoptosis. These findings provide experimental evidence supporting FA as a potential therapeutic candidate for POI.

## 1. Introduction

Premature ovarian insufficiency (POI) is a clinical syndrome characterized by the loss of ovarian function before the age of 40. It typically presents as menstrual disturbances (such as amenorrhea or oligomenorrhea) along with elevated gonadotropin levels and reduced estradiol levels [1,2,3]. Beyond infertility, POI significantly increases the risk of cardiovascular disease, osteoporosis, and neuropsychological disorders secondary to estrogen deficiency [2]. Although hormone replacement therapy can alleviate symptoms, it fails to restore ovarian function or fertility, and its long-term administration carries clear risks [4,5,6]. Therefore, exploring therapeutic strategies that can improve ovarian function while maintaining a favorable safety profile has become an urgent need in this field.

Cyclophosphamide (CTX) was the first chemotherapeutic agent demonstrated to be associated with POI and ovarian dysfunction [7]. In animal studies, CTX is a classic drug for inducing POI models [8] and causes ovarian oxidative stress (OS) [9]. OS is considered a key pathological contributor of POI [10]. OS not only directly impairs ovarian function [11]. but also induces endoplasmic reticulum (ER) stress, leading to abnormal activation of the unfolded protein response (UPR) [12,13,14]. Under sustained stress, the UPR can shift from an adaptive response to pro-apoptotic signaling, thereby affecting follicle development and granulosa cell survival [15]. Notably, ER stress is significantly upregulated in aging ovaries, suggesting its potential role in the pathological progression of POI [16,17].

Glucose-regulated protein 78 (Grp78) is a crucial molecular chaperone in the ER. Under homeostatic conditions, Grp78 binds to the UPR sensors, maintaining them in an inactive state. During ER stress, Grp78 dissociates from these sensors, thereby activating downstream signaling pathways. Consequently, the activation of Grp78 is widely regarded as a hallmark of the ER stress response [15]. Studies have shown that during follicle atresia, Grp78 expression is up-regulated and exceeds levels observed in healthy follicles [18]. This abnormal overexpression may be one of the important mechanisms leading to diminished ovarian reserve. When ER stress occurs, protein kinase R-like ER kinase (Perk) is released from the inhibitory effect of Grp78 and subsequently phosphorylating eukaryotic translation initiation factor 2α (eIF2α), thereby activating the Activating Transcription Factor 4 (ATF4)-C/EBP-Homologous Protein (CHOP) signaling axis and ultimately inducing apoptosis [15,19]. In ovarian tissue, excessive activation of ER stress is considered one of the critical factors contributing to ovarian damage [20]. This may be attributed to the increased apoptosis of granulosa cells (GCs) following the activation of the Perk pathway, which in turn affects normal follicle development and may even lead to follicle atresia [21,22]. Therefore, modulating dysregulated ER stress levels could serve as a potential therapeutic strategy for protecting ovarian function and delaying ovarian decline.

Ferulic acid (FA) is a natural phenolic compound widely used in the food and pharmaceutical industries [23]. It possesses multiple beneficial properties, including antioxidant, anti-inflammatory, and anti-platelet activities [24,25]. FA is also a major active component of several traditional Chinese medicinal herbs, such as *Angelica sinensis* (Oliv.) Diels [26] and *Ligusticum chuanxiong* Hort. [27]. Research indicates that FA is one of the primary components responsible for the blood-nourishing, menstrual regulation [28] and antioxidant effects [29] of these herbs. Notably, research indicates that *Ligusticum chuanxiong* Hort. can suppress ER stress-dependent apoptotic signaling [30], suggesting that FA, as its primary active component, may also possess ER stress-regulating potential. Furthermore, FA has been found to improve hormone profiles in women with impaired follicle development [31] and to promote bovine oocyte maturation and embryonic developmental potential [32]. These findings indicate that FA positively influences hormone balance, follicle development, and oocyte quality, aligning closely with the therapeutic goals for POI.

Unfortunately, direct evidence regarding the therapeutic efficacy of FA in POI remains limited. It is still unknown whether FA can exert protective effects on the ovaries by regulating ER stress. Notably, although FA exhibits clear antioxidant activity, whether it can more effectively protect ovarian function through the synergistic regulation of OS and ER stress is a scientific question that urgently requires in-depth exploration. Therefore, in this study, we used a CTX-induced POI mouse model [33] to assess the protective effects of FA on ovarian function. We also investigated the role of ER stress in FA’s mechanism of action, with a focus on the Perk-eIF2α-ATF4-CHOP signaling pathway. Simultaneously, using a GC damage model induced by 4-hydroperoxy cyclophosphamide (4-OHCP), the anti-apoptotic and ER stress-modulating effects of FA are further validated. These studies not only provide a new perspective for the clinical management of POI but also offer experimental evidence for the development of POI treatment strategies based on natural compounds.

## 2. Materials and Methods

### 2.1. Reagents

FA (Purity >99.5%) and sodium carboxymethyl cellulose (CMC-Na) were obtained from MedChemExpress (Monmouth Junction, NJ, USA), Dehydroepiandrosterone (DHEA) was sourced from APExBIO (Houston, TX, USA, B1375), and CTX was procured from Sigma-Aldrich (St. Louis, MO, USA, C0768-1G). The following assay kits were used: mouse follicle-stimulating hormone (FSH) ELISA kit (MU30265) and anti-Müllerian hormone (AMH) ELISA kit (MU30399) from Bioswamp (Wuhan, China); QuicKey Pro mouse estradiol (E_2_) ELISA kit (E-OSEL-M0008) from Elabscience (Wuhan, China); Cell Counting Kit-8 (CCK-8, 40203ES) and dihydroethidium (DHE, 50102ES) from Yeasen (Shanghai, China); ROS Fluorometric Assay Kit (Green, E-BC-K138-F) from Elabscience; Total Superoxide Dismutase (T-SOD) Assay Kit with WST-8 (S0101S) and Lipid Peroxidation Malondialdehyde (MDA) Assay Kit (S0131S) from Beyotime (Shanghai, China); and One-Step TUNEL In Situ Apoptosis Kit (Green, FITC, E-CK-A320) from Elabscience. 4-OHCP was purchased from Cayman (Ann Arbor, MI, USA, 19527), 4-Phenylbutyric acid (4-PBA) was obtained from Selleck (Houston, TX, USA, S3592), and tunicamycin (TM) was obtained from Selleck (S7894). Antibodies used in this experiment include: β-ACTIN (Proteintech, Wuhan, China, 20536-1-AP, 66009-1-Ig), AMH (Proteintech, 14461-1-AP), ATF4 (Proteintech, 60035-1-Ig), BAX (Proteintech, 60267-1-Ig), BCL-2 (Abcam, Cambridge, UK, ab182858), BCL-xL (Proteintech, 10783-1-AP), BMP15 (Proteintech, 18982-1-AP), Caspase 3 (Proteintech, 19677-1-AP), CHOP (Proteintech, 66741-1-Ig), eIF2α (Proteintech, 68479-1-Ig), GDF9 (Proteintech, 29309-1-AP), Grp78 (Proteintech, 11587-1-AP), p-eIF2α (CST, Danvers, MA, USA, 3398), p-Perk (Proteintech, 82534-1-RR), Perk (Proteintech, 68482-1-Ig), Biotin Conjugated AffiniPure Goat Anti-Rabbit IgG(H + L) (Boster, Wuhan, China, BA1003), DyLight 594 Conjugated AffiniPure Goat Anti-Rabbit IgG (H + L) (Boster, BA1142), Anti-rabbit IgG (H + L) (DyLight 800 4X PEG Conjugate) (CST, 5151), Anti-mouse IgG (H + L) (DyLight 680 Conjugate) (CST, 5470).

### 2.2. Animals and Treatment

Female C57BL/6 mice (6–8 weeks old, 18–20 g) were purchased from Gempharmatech Co., Ltd. (Nanjing, China), all mice are sexually mature and have no history of mating. The mice are housed in a specific pathogen-free (SPF) barrier environment, with 12 h of light/dark cycle, temperature 20~26 °C, humidity 40%~70%. The mice are provided with unrestricted access to food and water. All animal experiments have been approved by the Experimental Animal Center of Tongji Hospital Affiliated to Huazhong University of Science and Technology (ethics approval number: TJH-202308002).

Using a random number table method, mice were randomly assigned to five groups (*n* = 40 per group): control group, model group, low dose FA (LFA) group, high dose FA (HFA) group, and DHEA group. The LFA group and HFA group were given 40 mg/kg and 80 mg/kg of FA (referring to previous studies [34,35]), respectively, and the DHEA group was given 11.25 mg/kg of DHEA (corresponding to 75 mg/d for a 60 kg adult female [36]). All treatments were administered daily by oral gavage, starting on day 1 (D1). FA and DHEA were dissolved in physiological saline containing 0.5% CMC-Na; the control and model groups received the vehicle alone. Body weight was recorded daily throughout the 4-week gavage period. On D8, a single intraperitoneal injection of CTX (120 mg/kg) [33] was administered to all groups except the control to induce the POI model; control mice received an equivalent volume of physiological saline (10 mL/kg). Between D28 and D30, mice in the diestrous phase were euthanized. Serum and ovarian tissues were collected, and bilateral ovarian wet weights were measured. The ovary index was calculated as follows: combined ovarian wet weight (g)/body weight before euthanasia (g) × 100%.

### 2.3. Estrous Cycle Monitoring

Throughout the study, vaginal cytology smears were performed every morning. The stage of the estrous cycle was determined and recorded by an investigator blinded to the group through examination under a light microscope. The normal estrous cycle of mice consists of four distinct phases: diestrous, proestrous, estrous, and metestrous.

### 2.4. Ovarian Histology and Follicle Counting

Ovarian tissues were fixed in 4% paraformaldehyde for 24 h, dehydrated, and embedded in paraffin. Serial sections were cut at 5 μm thickness and mounted on glass slides. For histological evaluation, every fifth section was selected and stained with hematoxylin and eosin (H&E). Follicles at different developmental stages—primordial, primary, secondary, antral, and atretic—were identified and counted under an optical microscope. Counting was performed independently by experienced observers using standardized criteria. To ensure objectivity, only follicles containing a visible oocyte nucleus were included, and all samples were evaluated in a blinded manner.

### 2.5. Elisa Assay for Serum Hormone

Mouse blood samples were collected and left at room temperature for 2 h, then centrifuged at 3000 rpm for 15 min at 4 °C. Serum samples were stored at −80 °C pending assay. Serum levels of FSH, E_2_ and AMH were measured according to the steps in the Elisa kit instructions. Absorbance values at a wavelength of 450 nm were detected using microplate reader (Biotek, Winooski, VT, USA). The results were calculated using Origin 9.1 software.

### 2.6. Immunohistochemistry

Mouse ovaries were fixed in 4% paraformaldehyde, then dehydrated and paraffin-embedded. The embedded ovaries were sectioned at a thickness of 5 μm, and the sections were attached to slides. Sections were deparaffinized and hydrated, antigen repair was mediated by thermal repair method, and endogenous peroxidase activity was blocked by 3% H_2_O_2_. Subsequently, sections were blocked with 10% normal goat serum, followed by incubation with specific primary antibodies at 4 °C overnight. Next, the sections were incubated with goat anti-rabbit secondary antibody, DAB for color development, and hematoxylin for restaining. Finally, the slices were sealed and observed under a microscope.

### 2.7. Cell Culture

The granulosa cell line KGN cells were purchased from Cyagen Biosciences Inc. (Guangzhou, China) Granulosa cells were cultured in DMEM/F12 medium supplemented with 10% FBS and 100 U/mL penicillin and streptomycin at 37 °C in a 5% CO_2_ atmosphere. After cells attached, the control and model group continued to be cultured with complete medium, the treatment group was added with different concentrations of FA for 24 h, then 4-OHCP was added (except control). The ER stress inhibitor 4-PBA was added 2 h before 4-OHCP treatment, whereas the ER stress activator TM was administered 24 h prior to 4-OHCP exposure.

### 2.8. Cell Viability Assay

KGN cells (5 × 10^4^/mL) were seeded into 96-well plates. After cells attached, they were cultured with different concentrations of FA for 24 h, and then 4-OHCP was added (except control). Cell viability was detected using CCK8 kit, and the absorbance value at a wavelength of 450 nm was detected using microplate reader.

### 2.9. Oxidative Stress Detection

Fresh ovarian tissues were quick-frozen in liquid nitrogen, frozen sections were made, and the sections were stained with DHE reagent for 30 min in the dark, then observed under an inverted fluorescence microscope. Integrated fluorescence density was analyzed using ImageJ2. In vitro, using the ROS kit, KGN cells were stained to assess ROS production.

Ovarian homogenates and cell samples were collected, and the OS level of tissues and cells was detected using T-SOD and MDA kits according to the manufacturer’s instructions. Briefly, after lysis and centrifugation, WST-8/enzyme working solution or MDA detection working solution was added to the samples following the kit instructions. Following incubation or heating treatment, the absorbance was measured, and T-SOD activity or MDA content was calculated based on protein concentration.

### 2.10. Transcriptome Sequencing

Fresh mouse ovarian total RNA was extracted and quantified, and the quality of the RNA samples was determined using the Agilent 2100 Bioanalyzer (Santa Clara, CA, USA). The extracted RNA was used to construct transcriptome libraries with the Hieff NGS^®^ Ultima Dual-mode RNA Library Prep Kit (Yeasen), which were then sequenced on the DNBSEQ platform. After sequencing, invalid reads were filtered out, and the resulting clean reads were rapidly and accurately aligned to the reference genome using HISAT2 2.2.0. Differential expression analysis was performed using the DESeq2 package in R 4.4.2 to identify differentially expressed genes (DEGs, *p* value <0.05) between the two groups. The clusterProfiler package was then used to perform Gene Ontology (GO) and Kyoto Encyclopedia of Genes and Genomes (KEGG) pathway enrichment analyses on the DEG sets.

### 2.11. Molecular Docking

Obtain protein structures (ID: 8D1W, 4X7H) from the Protein Data Bank (PDB). Protein preprocessing was performed using Pymol 1.4.1 and AutoDock Tools 1.5.6, and the docking process between FA and the target protein was analyzed using AutoDock Tools 1.5.6. The binding energy was recorded and the docking results were visualized using Pymol software.

### 2.12. Molecular Dynamics Simulation

Molecular dynamics simulations were performed using Gromacs 2022. Force field parameters were obtained using Gromacs’ pdb2gmx tool and the AutoFF website. During simulations, the amber14sb force field was applied to the receptor protein’s molecular parameters, while the GAFF2 force field was used for the ligand’s molecular parameters. A 1 nm TIP3P cubic water box was added around the system for solvent simulation. Long-range electrostatic interactions were handled using the Particle Mesh Ewald (PME) method with a cutoff distance of 1 nm. Prior to molecular dynamics simulations, the system underwent energy optimization. The energy minimization process included 3000 steps of steepest descent optimization followed by 2000 steps of conjugate gradient optimization. Simulations were conducted under NPT conditions at 310 K and constant pressure, with a total simulation time of 100 ns.

### 2.13. Immunofluorescence

Cell climbing pieces were prepared, fixed in 4% paraformaldehyde, and then permeabilized with 0.5% Triton X-100 for 20 min at room temperature. The pieces were blocked with 10% normal goat serum, and then incubated with diluted primary antibody at 4 °C overnight. Next, the cells were washed with PBS, and then incubated with the fluorescent secondary antibody (594) at room temperature in the dark for 1 h. Finally, the nuclei were stained with DAPI, sealed with an anti-fluorescence quencher, and observed under an inverted fluorescence microscope (Olympus, Tokyo, Japan).

### 2.14. TUNEL

Cell climbing pieces were prepared, fixed in 4% paraformaldehyde, and then permeabilized with 0.5% Triton X-100 for 20 min at room temperature. Next, the pieces were incubated with TdT Equilibration Buffer at 37 °C for 20 min, and then with the labeling working solution at 37 °C for 60 min in the dark. Finally, the nuclei were stained with DAPI, sealed with an anti-fluorescence quencher, and observed under an inverted fluorescence microscope (Olympus, Tokyo, Japan).

### 2.15. Western Blot

Total protein from ovaries or GCs was extracted using RIPA lysis buffer containing protease and phosphatase inhibitors. Protein concentration was determined using a BCA protein assay kit. Proteins were separated by SDS-PAGE (8%, 10%) and then transferred to 0.22 μm PVDF membranes (Millipore, Burlington, MA, USA). The membranes were blocked with 5% skim milk or 3% BSA (bovine serum albumin, for phosphorylated proteins blocking) at room temperature for 2 h, and then incubated with the primary antibody at 4 °C overnight. Next day, the membranes were washed 3 times with TBST and incubated with the appropriate secondary antibody for 2 h, followed by three more washes with TBST. Images were visualized using the Odyssey infrared imaging system (LI-COR, Lincoln, NE, USA) and analyzed using ImageJ2.

### 2.16. Real-Time Quantitative PCR

Tissue and cell RNA was extracted using TRIZOL reagent (TaKaRa Bio, Shiga, Japan) according to the protocol provided in the kit. Nano Drop software was used to determine total RNA concentration. Reverse transcription was performed using the PrimeScript RT reagent kit (TaKaRa Bio, Shiga, Japan) to obtain cDNA. Real-time qPCR was then performed using the LightCycler^®^ 96 system (Roche Diagnostics, Basel, Switzerland) according to the instructions of SYBR Green Master Mix kit (TaKaRa Bio, Shiga, Japan). Relative gene expression was calculated using the 2^−ΔΔCt^ method. The animal and cellular primer sequences used in the present study are shown in Table 1 and Table 2, respectively.

### 2.17. Data Analysis

All data in the present study are from at least three independent experiments. GraphPad Prism software 8.0 was used for statistical analysis. One-way analysis of variance (ANOVA) was used, followed by Tukey’s post hoc test, to compare the mean differences between multiple groups. Where data violated the assumptions for parametric analysis, the Kruskal–Wallis test was applied. All data are expressed as mean ± standard error (SEM), and *p* < 0.05 was considered statistically significant.

## 3. Results

### 3.1. FA Maintains Normal Estrous Cycle, Restores Body Weight and Ovarian Index in POI Mice

**Figure 1:**
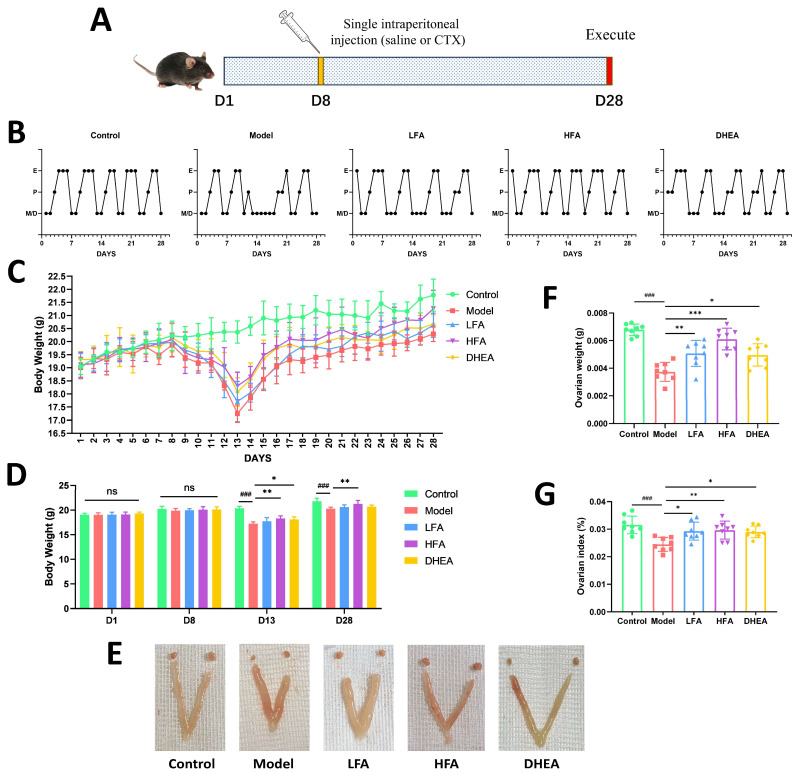
FA improves the estrous cycle, ovary and body weights of POI mice. (**A**) Schematic diagram of animal experiment processing. (**B**) Estrous cycle of each group. (**C**) Dynamic changes in body weight of mice in each group over 28 days (*n* = 6). (**D**) Comparison of body weight in each group on D1, D8, D13 and D28 (*n* = 6). (**E**) Representative images of the morphology of the ovaries and uterus in each group. (**F**) Wet weight of ovaries in each group (*n* = 8). (**G**) Ovarian index in each group (*n* = 8). ^###^ *p* < 0.001 vs. control group; * *p* < 0.05, ** *p* < 0.01, *** *p* < 0.001 vs. model group; ns, no significant differences were observed among the groups. E, estrus; P, proestrus; M, metestrus; D, diestrus.

A schematic diagram of the animal experimental protocol is summarized in Figure 1A. DHEA, which is clinically used to improve ovarian reserve and responsiveness [37,38], was included as a positive control. Figure 1B illustrates the dynamic changes in the estrous cycle across groups. Within 3–4 days after CTX injection, the model group displayed marked estrous cycle disruption, characterized by shortened or absent estrous phases and prolonged diestrous. In contrast, mice in the LFA, HFA, and DHEA groups continued to exhibit regular estrous phases, with the HFA group maintaining largely stable cyclicity. No significant differences in body weight were observed among groups at day 1 (D1) or D8. Following CTX administration, all non-control groups experienced a gradual decline in body weight, reaching the lowest point at D13, followed by gradual recovery. The model group showed the most pronounced weight loss at D13 and D28, which was significantly attenuated in the HFA group (Figure 1C,D). Representative images of ovarian and uterine morphology are presented in Figure 1E and Figure S1. Ovarian size was markedly reduced in the model group, whereas FA treatment ameliorated this atrophy. DHEA also conferred some improvement in ovarian morphology, although unilateral ovarian atrophy was noted in some cases. Consistent with these observations, both ovarian wet weight and ovarian index were significantly lower in the model group than in controls, but were restored by FA or DHEA treatment (Figure 1F,G).

### 3.2. FA Improves Sex Hormone Levels in POI Mice

**Figure 2:**
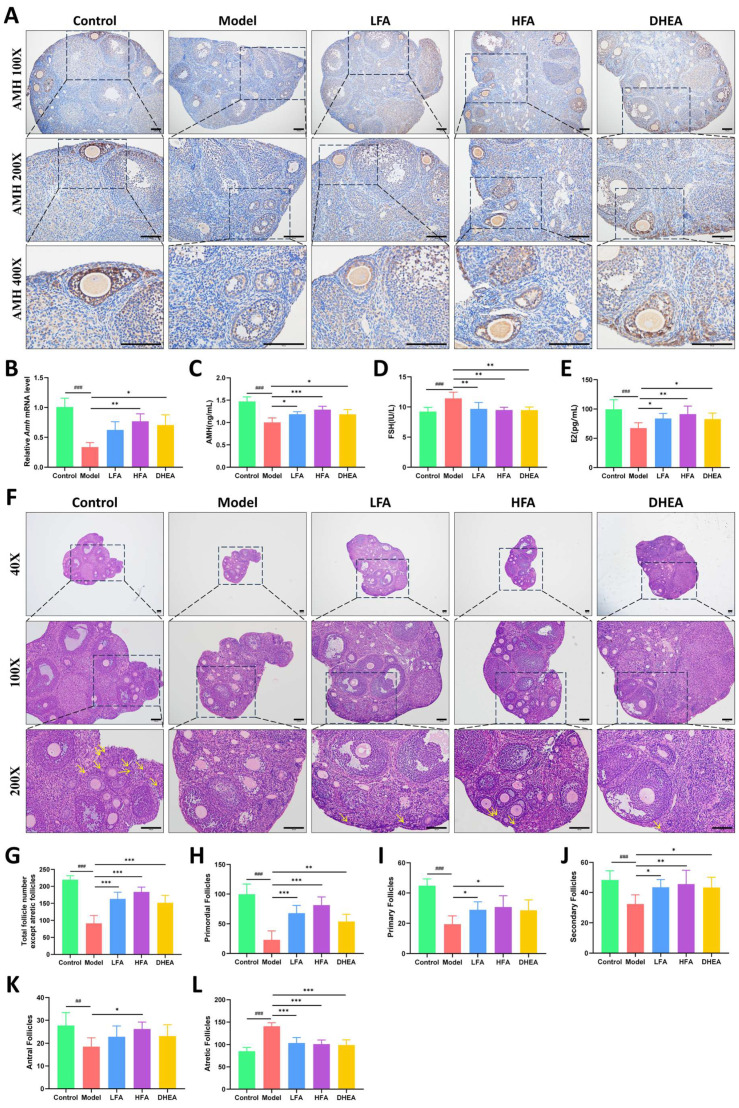
Effect of FA on ovarian morphology, follicle number, and serum hormone levels in POI mice. (**A**) Representative ovarian AMH immunohistochemical images for each group, scale bar = 100 μm. (**B**) Relative expression of *Amh* mRNA in the ovaries of each group (*n* = 6). (**C**–**E**) Serum AMH, FSH, and E_2_ levels, respectively (*n* = 6–8). (**F**) Representative images of ovarian sections stained with HE, with yellow arrows indicating primordial follicles, scale bar = 100 μm. (**G**–**L**) Statistical results for the number of total follicles (excluding atretic follicles), primordial follicles, primary follicles, secondary follicles, antral follicles, and atretic follicles (*n* = 7–9). ^##^ *p* < 0.01, ^###^ *p* < 0.001 vs. control group; * *p* < 0.05, ** *p* < 0.01, *** *p* < 0.001 vs. model group.

AMH reflects ovarian reserve. The immunohistochemical staining results for AMH in the ovaries of each group are shown in Figure 2A. AMH is mainly expressed in the preantral follicles of the mice. Compared with the control group, AMH expression was significantly reduced in the model group. Furthermore, serum AMH and ovarian *Amh* mRNA level was also significantly reduced in the model group, while FA and DHEA treatment improved these AMH levels (Figure 2A–C). In addition, the present study indicated higher serum FSH levels and lower E_2_ levels in the model group (Figure 2D,E). Compared to the model group, FA and DHEA treatment significantly decreased FSH levels and increased E_2_ levels (Figure 2D,E).

### 3.3. FA Restores Ovarian Morphology and Follicle Development in POI Mice

Figure 2F shows whole ovarian sections and localized magnified views of each group. In the model group, a significant reduction in ovarian size and presence of damaged follicles were observed, as indicated by abnormal follicle morphology, loose arrangement of GCs, and a decrease in growing follicles at all stages, which were replaced by severe fibrosis of the ovarian stroma. As shown in Figure 2G, the total number of follicles (excluding atretic follicles) in the model group was significantly lower than in the other groups, indicating severe follicle loss in the model group. Compared with the control group, the number of primordial follicles was significantly reduced in the model group, whereas FA and DHEA pretreatment maintained ovarian reserve, especially in the HFA group (Figure 2H). Significant differences were also observed in the number of primary, secondary, and antral follicles between the model and control group. FA treatment reversed this damage in a dose-dependent manner (Figure 2I–K). While the number of growing follicles increased in the DHEA group, but only secondary follicles showed a statistically significant difference (Figure 2I–K). In addition, the number of atretic follicles was significantly increased in the model group compared with the control group, and treatment with FA or DHEA reduced this count (Figure 2L).

**Figure 3:**
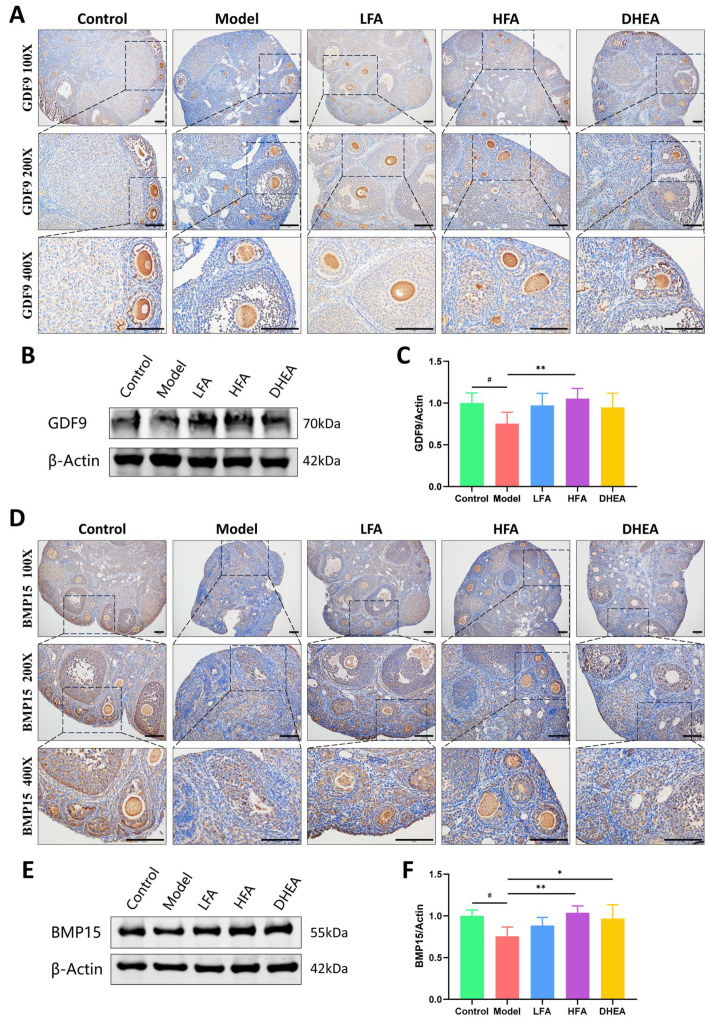
Effect of FA on the expression of follicle development-related factors in POI mice. Representative immunohistochemical images of GDF9 (**A**) and BMP15 (**D**) of each group, scale bar = 100 μm. Representative images of GDF9 (**B**) and BMP15 (**E**) protein bands in the ovaries of each group. Statistical analysis of GDF9 (**C**) and BMP15 (**F**) protein expression in each group (*n* = 5–7). ^#^ *p* < 0.05 vs. control group; * *p* < 0.05, ** *p* < 0.01 vs. model group.

Ovarian follicle development is regulated by complex factors, among which growth differentiation factor 9 (GDF9) and bone morphogenetic protein 15 (BMP15) of the TGF-β family have been shown to be essential. As shown in Figure 3A,D, GDF9 is mainly expressed in oocytes, while BMP15 is positively stained in both oocytes and GCs. Compared with the control group, the expression of GDF9 and BMP15 was significantly reduced in the model group, which was consistent with the Western blot results (Figure 3B,C,E,F). HFA treatment substantially restored the expression of both factors (Figure 3B,C,E,F), with a particularly pronounced effect on GDF9. In the DHEA group, a significant increase was observed in BMP15 expression, whereas the change in GDF9 was not statistically significant. Although LFA treatment led to upward trends in both GDF9 and BMP15, the differences did not reach statistical significance.

Taken together, the results of serum hormone, follicle counts and follicle development factors indicate that HFA treatment has a positive effect on maintaining ovarian reserve and improving follicle development.

### 3.4. FA Reduces Oxidative Stress in POI Mouse Ovaries

**Figure 4:**
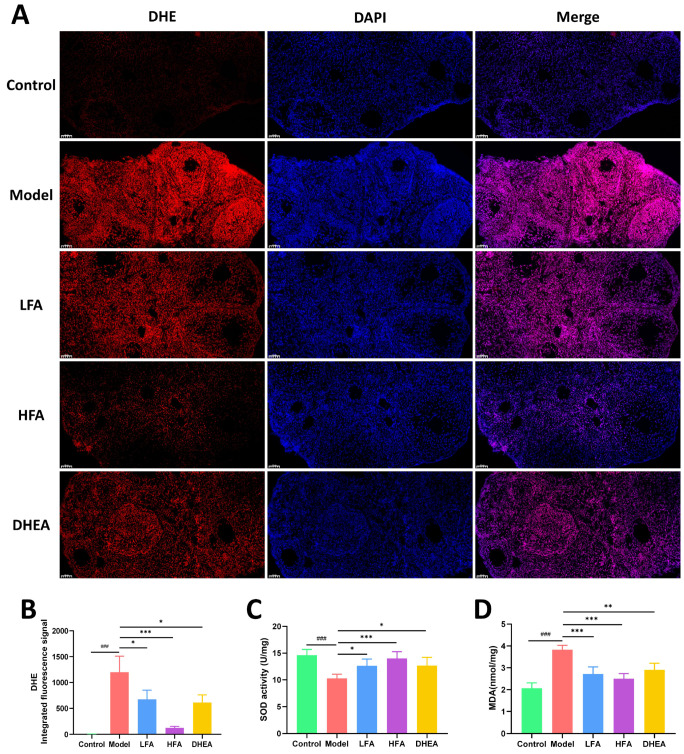
Effect of FA on oxidative stress levels in the ovaries of POI mice. (**A**,**B**) Representative images of ovarian frozen sections stained with DHE for each group, scale bar = 100 μm. (**B**) Statistical analysis of DHE (*n* = 3). (**C**) Statistical analysis of T-SOD activity (*n* = 6). (**D**) Statistical analysis of MDA (*n* = 6). ^###^ *p* < 0.001 vs. control group; * *p* < 0.05, ** *p* < 0.01, *** *p* < 0.001 vs. model group.

The ROS levels in mouse ovaries were assessed by DHE staining. As shown in Figure 4A,B, the ovarian ROS levels in the model group were significantly higher than those in the control group. Positive staining was observed in ovarian GCs and ovarian stroma. FA and DHEA treatment significantly reduced ROS levels in the ovaries, with the most significant improvement observed in the HFA group. In addition, the ovarian MDA (a biomarker of lipid or protein peroxidation) level of the model group was significantly higher, while T-SOD (an antioxidant enzyme) level was significantly lower (Figure 4C,D). In contrast to the model group, FA and DHEA treatment caused a significant decrease in MDA level and a significant increase in T-SOD level (Figure 4C,D), indicating that FA and DHEA help to reduce the oxidative level in the ovaries of POI mice.

### 3.5. Screening for FA-Regulated Targets in POI Mouse Ovaries Using Transcriptome Sequencing

**Figure 5:**
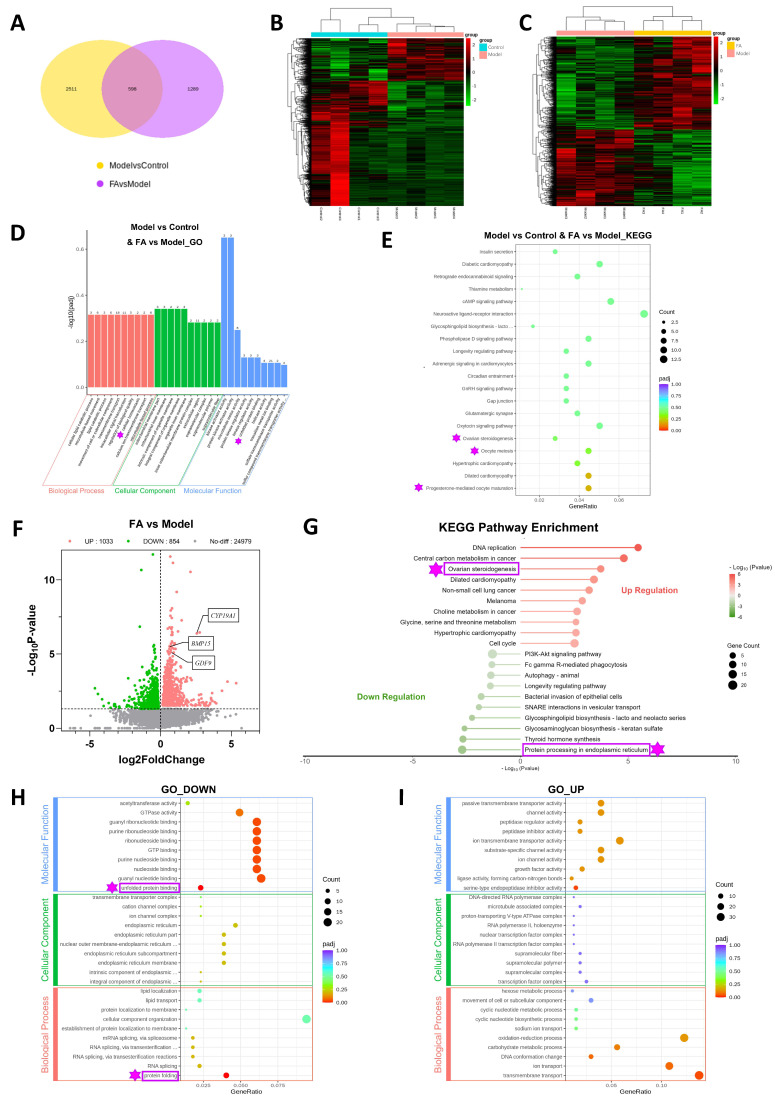
Screening of FA-regulated sites in POI mouse ovaries using transcriptome sequencing. (**A**) Venn diagram of DEGs between the model group and the control group, and between the FA group and the model group. (**B**) Heat map of DEGs between the model group and the control group. Red indicates upregulation, green indicates downregulation (*n* = 4). (**C**) Heat map of DEGs between the FA group and the model group. Red indicates upregulation, green indicates downregulation (*n* = 4). (**D**) GO enrichment analysis for Model vs. Control and FA vs. Model. The height of the bar indicates the size of the difference, and the number represents the number of DEGs. (**E**) KEGG enrichment pathway analysis for Model vs. Control and FA vs. Model. Dot color indicates the significance of the difference, and dot size represents the number of DEGs. (**F**) Volcano plot of genes in the FA group vs. the model group. Red indicates upregulation, green indicates downregulation, and gray indicates no difference. (**G**) KEGG enriched pathways of up- and downregulated genes in the FA group vs. the model group, with the top 10 shown for each. Red indicates upregulation, green indicates downregulation, and darker colors indicate more significant differences. The size of the dots represents the number of DEGs. (**H**) GO enrichment analysis of downregulated genes in the FA group vs. the model group. Dot color indicates the significance of the difference, and dot size represents the number of DEGs. (**I**) GO enrichment analysis of upregulated genes in the FA group vs. the model group. Dot color indicates the significance of the difference, and dot size represents the number of DEGs.

To identify the precise biological mechanisms by which FA regulates and enhances ovarian function in POI mice, ovarian samples were subjected to transcriptome sequencing. The HFA group was selected to represent the FA treatment group for this analysis. As illustrated in Figure 5A, a total of 3109 differentially expressed genes (DEGs) were identified in the model group compared to the control group (Supplementary Table S1), while 1887 DEGs were identified in the FA group compared to the model group (Supplementary Table S2). Among the three groups, 598 common DEGs were identified. The DEG heatmaps comparing the control versus model groups and the model versus FA groups are shown in Figure 5B and Figure 5C, respectively, with green indicating downregulation and red indicating upregulation. Functional enrichment analysis of the 598 common DEGs among the three groups revealed that the groups primarily differed in the following biological processes: progesterone-mediated oocyte maturation, oocyte meiosis, ovarian steroidogenesis, intracellular homeostasis, and unfolding protein binding (Figure 5D,E).

To further elucidate the molecular targets of FA on POI, we analyzed differential gene expression between FA-treated and model groups. The volcano plot identified 1033 up-regulated and 854 down-regulated genes in the FA group (Figure 5F and Supplementary Table S2). Notably, FA significantly increased the expression of key folliculogenesis genes (*Gdf9*, *Bmp15*) and the steroidogenic gene *Cyp19a1* (Figure 5F), consistent with the observed improvements in follicle development and hormone levels in FA-treated mice. KEGG pathway analysis demonstrated that FA treatment downregulated protein processing in the ER and upregulated the ovarian steroidogenesis pathway (Figure 5G and Supplementary Table S3). GO analysis further indicated downregulation of protein folding, unfolded protein binding, and ER-related cellular components (Figure 5H and Supplementary Table S4), along with upregulation of growth factor activity in FA-treated ovaries (Figure 5I and Supplementary Table S5). Collectively, these findings suggest that POI mice may exhibit disrupted ER homeostasis and a state of cellular stress. FA treatment appears to alleviate ER stress while concurrently promoting follicle development and steroidogenic pathways, thereby cooperatively improving ovarian function. This transcriptomic evidence supports a mechanism by which FA protects ovarian function through modulation of ER homeostasis and the follicle microenvironment.

### 3.6. Molecular Docking and Dynamics Simulations of FA with Grp78 and Perk

**Figure 6:**
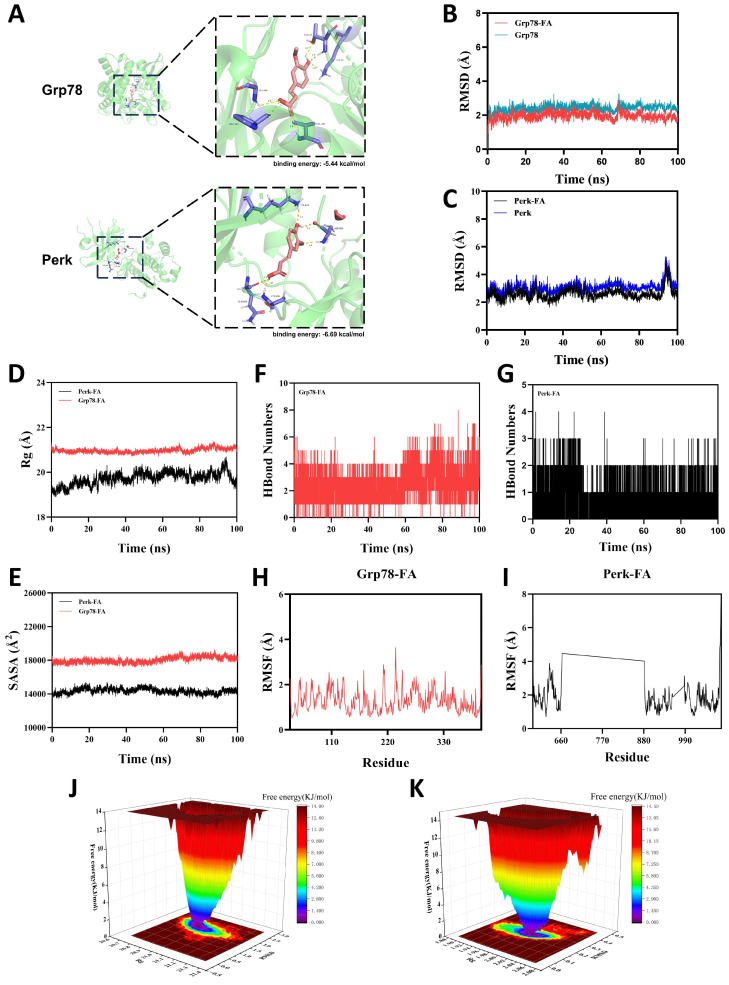
Molecular docking and molecular dynamics simulation of FA with Grp78 and Perk. (**A**) Molecular docking results of FA with Grp78 and Perk. (**B**,**C**) RMSD values for Grp78-FA and Perk-FA complexes over time. (**D**) Rg values for Grp78-FA and Perk-FA complexes over time. (**E**) SASA values for Grp78-FA and Perk-FA complexes over time. (**F**,**G**) HBonds values for Grp78-FA and Perk-FA complexes over time. (**H**,**I**) RMSF values for Grp78-FA and Perk-FA complexes. (**J**,**K**) Free energy landscape plots.

Based on the transcriptomic analysis results, we further evaluated the interaction between FA and the ER stress-related proteins Grp78 and Perk using molecular docking. The results showed that FA exhibits strong binding to both targets, with calculated binding energies of −5.44 kcal/mol for Grp78 and −6.69 kcal/mol for Perk (Figure 6A).

To evaluate the stability of these complexes, molecular dynamics simulations were conducted. The root mean square deviation (RMSD) was used to monitor conformational stability of proteins and ligands. As shown in Figure 6B, the Grp78 and Grp78-FA systems reached equilibrium after 80 ns, stabilizing around 2.3 Å and 1.7 Å, respectively. The Perk-FA complex exhibited slight fluctuations during motion, oscillating around 3 Å overall (Figure 6C). Together, these data indicate stable binding of FA to both Grp78 and Perk.

The radius of gyration (Rg) characterizes overall structural changes and reflects the compactness of protein structures. The Grp78-FA complex exhibited stable Rg fluctuations, while the Perk-FA complex system showed minor fluctuations during simulation (Figure 6D). Solvent-accessible surface area (SASA) measures the proportion of a protein surface exposed to water, aiding in predicting conformational changes during interactions. Throughout the simulation, both Grp78-FA and Perk-FA complexes exhibited slight fluctuations in SASA (Figure 6E), suggesting that FA binding may influence the protein’s binding microenvironment and induce some degree of SASA variation.

Hydrogen bonds play a crucial role in ligand-protein interactions. The number of hydrogen bonds between Grp78 and FA ranged from 0 to 8, with approximately 4 hydrogen bonds present in most cases (Figure 6F). The number of hydrogen bonds between Perk and FA ranged from 0 to 4, with approximately 2 hydrogen bonds present in most cases (Figure 6G). This indicates that both Grp78-FA and Perk-FA complexes exhibit robust hydrogen bonding interactions.

Root mean square fluctuation (RMSF) reflects the flexibility of amino acid residues within proteins. As shown in Figure 6H,I, the Grp78-FA complex exhibits relatively low RMSF values (mostly below 2.4 Å), while the Perk-FA complex also shows relatively low RMSF values (mostly below 3 Å). Consequently, both complexes demonstrate low flexibility and high stability.

The Free Energy Landscape (FEL) depicts the free energy distribution calculated from RMSD and Rg during protein-ligand molecular dynamics simulations. Figure 6J,K illustrate the simulated dynamic processes of the Grp78-FA and Perk-FA complexes, respectively. A color gradient represents free energy levels, decreasing from red (high energy) to blue (low energy).

Collectively, these computational results demonstrate that the Grp78-FA and Perk-FA complex systems exhibit stable binding with well-defined hydrogen bonding interactions. This indicates that FA can potentially interact with the target proteins Grp78 and Perk, providing a structural basis for the potential ability of FA to modulate the ER stress pathway.

### 3.7. FA Inhibits Perk/eIF2α/ATF4/CHOP Pathway in POI Mouse Ovaries

**Figure 7:**
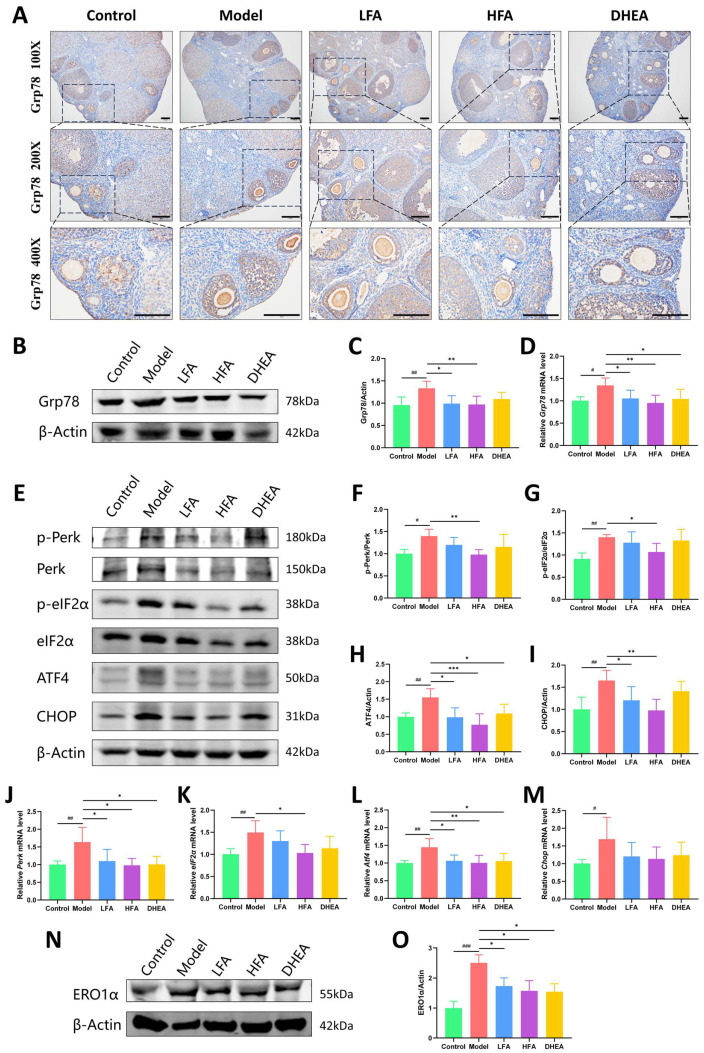
Effect of FA on Grp78 expression and on the Perk/eIF2α/ATF4/CHOP pathway in POI mice. (**A**) Representative immunohistochemical images of Grp78 of each group, scale bar = 100 μm. (**B**) Representative images of Grp78 protein bands in the ovaries of each group. (**C**) Statistical analysis of Grp78 protein expression in each group (*n* = 5–6). (**D**) Relative expression of *Grp78* mRNA in ovaries of each group (*n* = 6). (**E**) Representative images of ovarian p-Perk, Perk, p-eIF2α, eIF2α, ATF4, and CHOP protein bands in each group. (**F**–**I**) Statistical analysis of p-Perk/Perk, p-eIF2α/eIF2α, ATF4, and CHOP expression in each group (*n* = 5–8). (**J**–**M**) Relative expression of *Perk*, *eIF2α*, *Atf4*, and *Chop* mRNA in the ovaries of each group (*n* = 6). (**N**) Representative images of ovarian ERO1α protein bands in each group. (**O**) Statistical analysis of ERO1α expression in each group (*n* = 3). ^#^ *p* < 0.05, ^##^ *p* < 0.01, ^###^ *p* < 0.001 vs. control group; * *p* < 0.05, ** *p* < 0.01, *** *p* < 0.001 vs. model group.

The activation of Grp78 serves as a marker of the ER stress response. Immunohistochemical analysis detected Grp78 expression in the ovarian tissues of all groups (Figure 7A), primarily localized to the GCs. Ovaries from the control group showed low Grp78 expression, whereas the model group exhibited a marked increase. This elevated expression was attenuated in both FA and DHEA treated groups. Meanwhile, the Grp78 protein and mRNA levels were significantly higher in the model group than in the control group, and both LFA and HFA treatments significantly reduced Grp78 at both the protein and transcript levels (Figure 7B–D). In the DHEA group, a significant decrease was observed only in *Grp78* mRNA (Figure 7B–D).

Furthermore, the model group showed significant upregulation of key ER stress-related proteins, including the p-Perk/Perk and p-eIF2α/eIF2α ratios, as well as ATF4, and CHOP expression, compared with the control group. FA treatment, particularly at the high dose (HFA), effectively reversed these elevations (Figure 7E–I). DHEA significantly suppressed ATF4 protein expression. However, it did not significantly affect the p-Perk/Perk and p-eIF2α/eIF2α ratios or CHOP protein levels. A similar trend was observed at the transcript level, with mRNA expression of *Perk*, *eIF2a*, *Atf4* and *Chop* being significantly increased in the model group. HFA significantly reduced *Perk*, *eIF2a* and *Atf4* mRNA levels. Although *Chop* expression also decreased, the change was not statistically significant (Figure 7J–M). It is noteworthy that, compared to the model group, the DHEA group exhibited significant decreases only in the mRNA levels of *Perk* and *Atf4* (Figure 7J,L), suggesting that the effect of DHEA on this pathway may be limited.

ER oxidoreductase 1α (ERO1α), a key downstream target of ATF4, is a crucial regulator of intracellular OS. In this study, we further found that ERO1α expression was significantly elevated in the ovaries of POI model mice, while both FA and DHEA treatments effectively reduced its expression (Figure 7N,O). This suggests that FA may suppress the ATF4-ERO1α axis, thereby blocking the further increase in intracellular oxidative pressure caused by excessive ERO1α activity and consequently alleviating the oxidative damage state in POI ovaries.

### 3.8. FA Reduces 4-OHCP-Induced KGN Cell Damage and Oxidative Stress

**Figure 8:**
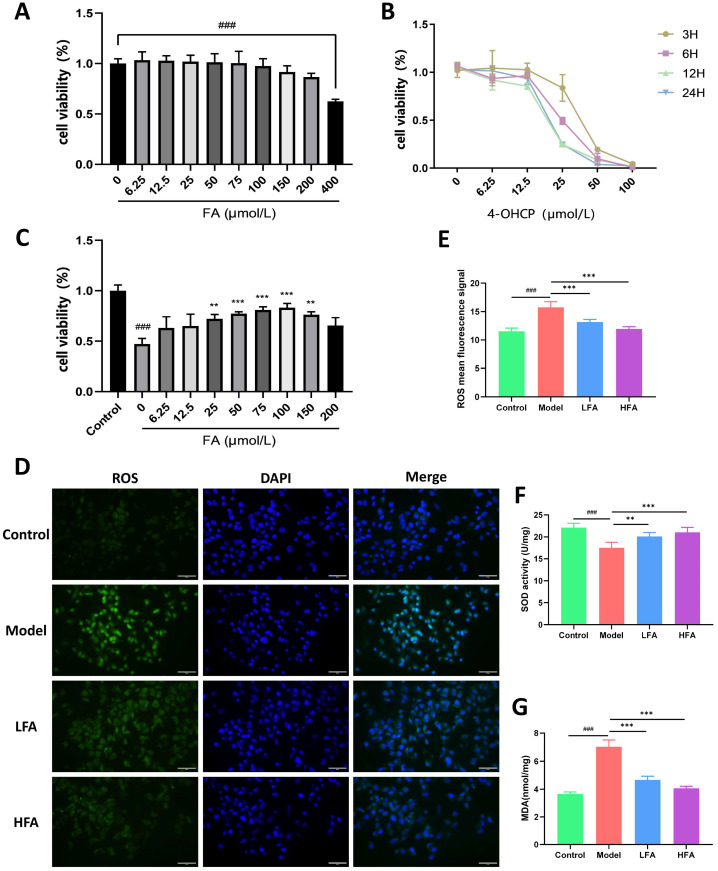
Effect of FA on the viability and oxidative stress of 4-OHCP-damaged KGN cells. (**A**) Cell viability after 24 h of treatment with different concentrations of FA (*n* = 3). (**B**) Cell viability after treatment with different concentrations of 4-OHCP for different durations (*n* = 3). (**C**) Cell viability after pretreatment with different concentrations of FA for 24 h, followed by treatment with 25 μM 4-OHCP for 6 h (*n* = 3). (**D**,**E**) Representative fluorescence images and statistical analysis of ROS in each group, scale bar = 50 μm. (**F**) Statistical analysis of T-SOD activity in cells (*n* = 5). (**G**) Statistical analysis of MDA in cells (*n* = 4–5). ^###^ *p* < 0.001 vs. control group; ** *p* < 0.01, *** *p* < 0.001 vs. model group.

To determine the appropriate drug concentration, KGN cells were first treated with varying concentrations of FA. As shown in Figure 8A, FA concentrations ranging from 6.25 to 200 μmol/L had no significant effect on cell viability, whereas FA at 400 μmol/L caused a significant inhibition of KGN proliferation. Since 4-OHCP is the active metabolite of cyclophosphamide in vivo, it was used for the in vitro injury model. Cells were treated with different concentrations of 4-OHCP for various durations; the condition resulting in approximately 50% cell viability (25 μmol/L for 6 h; Figure 8B) was selected for subsequent experiments. Following FA pretreatment and subsequent 4-OHCP exposure, cell viability of the model group (0 μmol/L FA) was significantly reduced compared with the control group, while 100 μmol/L FA most effectively maintain cell viability (Figure 8C). Consequently, 50 and 100 μmol/L FA were selected as the LFA and HFA concentrations, respectively, for further studies.

As shown in Figure 8D,E, the ROS levels in the model group were significantly higher than those in the control group, whereas LFA and HFA pretreatment significantly reduced ROS production. In addition, compared with the control group, model cells exhibited significantly increased MDA and decreased T-SOD levels (Figure 8F,G). Both LFA and HFA restored the balance between these two markers in KGN cells (Figure 8F,G).

### 3.9. FA Alleviates ER Stress in KGN Cells by Modulating the Perk/eIF2α/ATF4/CHOP Pathway

**Figure 9:**
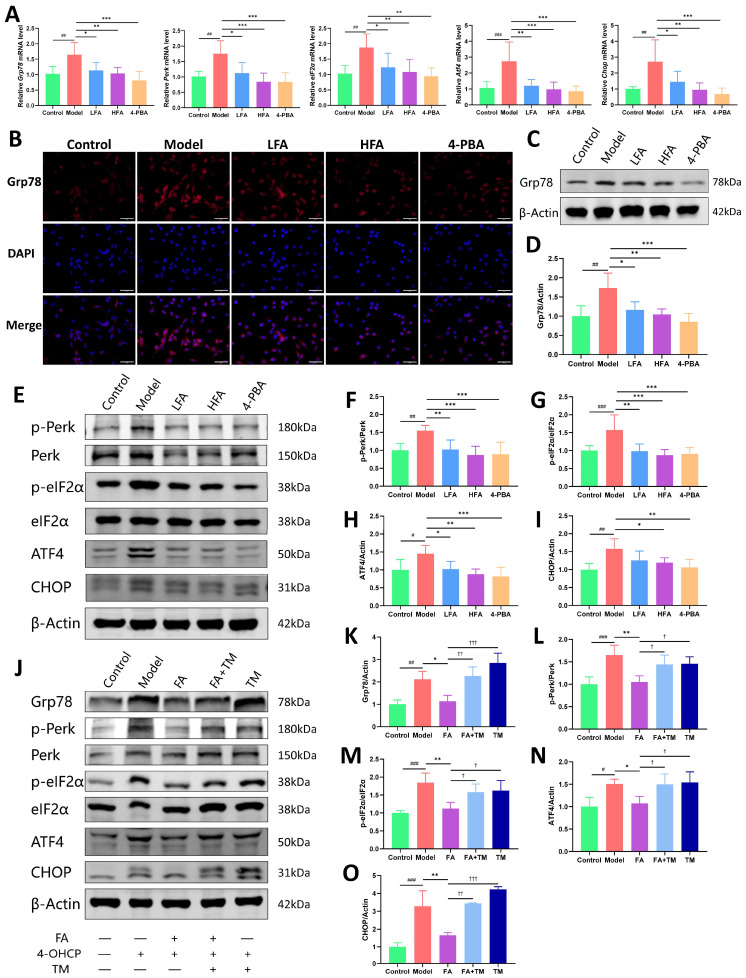
Effect of FA on Grp78 and the Perk/eIF2α/ATF4/CHOP pathway in 4-OHCP-damaged KGN cells. (**A**) Relative expression of *Grp78*, *Perk*, *eIF2α*, *Atf4*, and *Chop* mRNA in cells of each group (*n* = 5–7). (**B**) Representative immunofluorescence images of Grp78 of each group, scale bar = 50 μm. (**C**) Representative images of Grp78 protein bands of each group. (**D**) Statistical analysis of Grp78 protein expression of each group (*n* = 5). (**E**) Representative images of p-Perk/Perk, p-eIF2α/eIF2α, ATF4, and CHOP protein bands in the indicated groups. (**F**–**I**) Statistical analysis of the protein levels shown in (**E**) (*n* = 5–8). (**J**) Representative images of Grp78, p-Perk/Perk, p-eIF2α/eIF2α, ATF4, and CHOP protein bands in KGN cells treated with or without TM. (**K**–**O**) Statistical analysis of the protein levels shown in (**J**) (*n* = 3–5). ^#^ *p* < 0.05, ^##^ *p* < 0.01, ^###^ *p* < 0.001 vs. control group; * *p* < 0.05, ** *p* < 0.01, *** *p* < 0.001 vs. model group; ^†^ *p* < 0.05, ^††^ *p* < 0.01, ^†††^ *p* < 0.001 vs. FA group.

To investigate the regulatory effect of FA on ER stress in KGN cells, we examined the expression of key ER stress markers, using the ER stress inhibitor 4-PBA (1 mM [39]) as a positive control. As shown in Figure 9A, cells of the model group showed a significant increase in *Grp78*, *Perk*, *eIF2α*, *Atf4* and *Chop* mRNA compared with the control group, while FA and 4-PBA treatment reversed this abnormal increase. Consistent with the mRNA findings, Grp78 protein expression was also markedly elevated in the model group and subsequently attenuated by FA or 4-PBA administration (Figure 9B,D). Furthermore, the model group showed increased p-Perk/Perk and p-eIF2α/eIF2α ratios, along with upregulation of ATF4 and CHOP proteins (Figure 9E–I). While both 4-PBA and HFA treatment significantly suppressed the activation of these proteins, LFA treatment significantly reduced p-Perk/Perk, p-eIF2α/eIF2α, and ATF4 levels but did not significantly affect CHOP expression (Figure 9E–I).

To further validate these findings, cells were treated with the ER stress activator TM (1 μM [40]). As shown in Figure 9J–O, the FA + TM and TM groups displayed significantly elevated levels of Grp78, ATF4, and CHOP, as well as increased p-Perk/Perk and p-eIF2α/eIF2α ratios compared to the FA group. This reversal suggests that the inhibitory effects of FA on the ER stress pathway were counteracted by TM.

### 3.10. FA Inhibits 4-OHCP-Induced Apoptosis in KGN Cells

**Figure 10:**
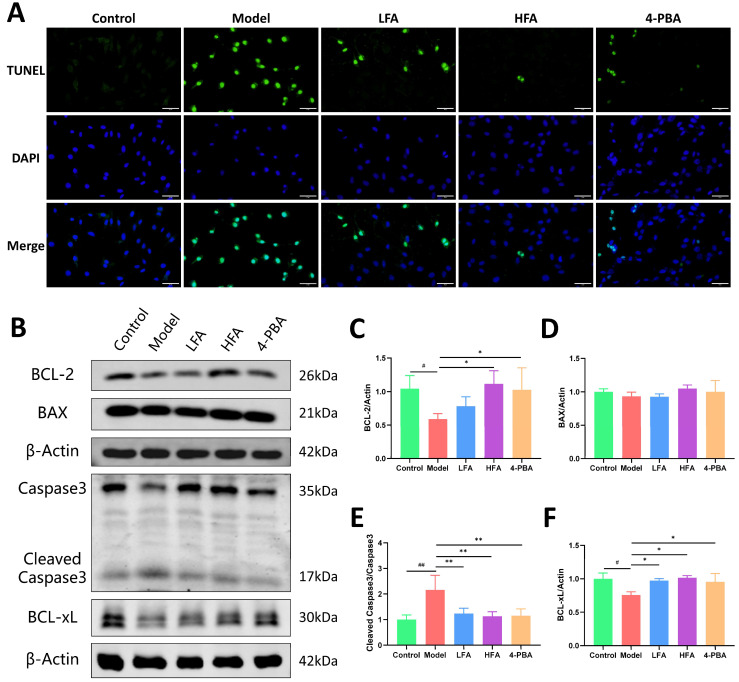
Effect of FA on apoptosis of 4-OHCP-damaged KGN cells. (**A**) Representative immunofluorescence images of TUNEL in each group, scale bar = 50 μm. (**B**) Representative images of BCL-2, BAX, cleaved Caspase3, Caspase3 and BCL-xL protein bands of each group. (**C**–**F**) Statistical analysis of BCL-2, BAX, cleaved Caspase3/Caspase3 and BCL-xL protein expression in cells of each group (*n* = 3–5). ^#^ *p* < 0.05, ^##^ *p* < 0.01 vs. control group; * *p* < 0.05, ** *p* < 0.01 vs. model group.

As shown in Figure 10A, minimal apoptosis was observed in control KGN cells under normal culture conditions. In contrast, 4-OHCP exposure markedly increased apoptotic cell numbers. This effect was significantly ameliorated by both LFA and HFA treatment, as well as by the ER stress inhibitor 4-PBA, with HFA exhibiting the most pronounced protective effect. Further analysis of apoptosis-related proteins revealed that the model group exhibited a significant increase in the cleaved Caspase 3/Caspase 3 ratio and a decrease in the anti-apoptotic proteins BCL-2 and BCL-xL (Figure 10B,C,E,F). These changes were reversed by HFA, and 4-PBA treatments, which significantly reduced Caspase 3 activation and increased BCL-2 and BCL-xL expression. However, BAX protein expression did not differ significantly among groups (Figure 10D). LFA treatment also significantly decreased the cleaved Caspase 3/Caspase 3 ratio and raised BCL-xL levels, although its effect on BCL-2 was not statistically significant. Above all, the results demonstrate that FA may effectively suppress 4-OHCP-induced apoptosis in KGN cells.

## 4. Discussion

This study systematically investigated the protective effects of FA on a CTX-induced POI model and elucidated its underlying mechanisms. Our results demonstrated that FA not only significantly improved ovarian function indices, restored endocrine balance, and promoted follicle development in POI mice but also effectively alleviated OS, reduced Grp78 expression, and inhibited the activation of the Perk/eIF2α/ATF4/CHOP pathway. These findings provide important experimental evidence supporting the potential therapeutic use of FA for POI and reveal that its protective effects are likely achieved through the coordinated regulation of OS and ER stress.

POI is characterized by accelerated primordial follicle depletion leading to ovarian dysfunction [41]. In this study, CTX—a non-cell-cycle-specific agent known to induce dose-dependent ovarian damage [42,43]—was used to establish a POI mouse model. CTX not only damages quiescent follicles but also severely impairs growing follicles. The model mice exhibited marked ovarian atrophy, reduced primordial and developing follicles, increased follicle atresia, and interstitial fibrosis, confirming severe ovarian reserve impairment. FA treatment effectively counteracted these changes, preserving primordial follicles, reducing atresia and fibrosis, and attenuating the progression of POI.

FSH promotes preantral follicle development and estrogen synthesis [44,45], while AMH reflects the growing follicle pool and inhibits primordial follicle recruitment [46]. AMH not only reflects the dynamic ovarian reserve but also influences the innate pool of follicles, making it a widely used marker for assessing remaining ovarian reserve [46,47,48]. In this study, POI mice showed elevated FSH and decreased E_2_ and AMH levels. FA treatment reversed these hormonal imbalances, significantly increasing serum AMH, indicating improved ovarian reserve. As both E_2_ and AMH are primarily produced by granulosa cells of developing follicles [46,49], the rise in developing follicles following FA treatment further explains the restoration of these hormone levels.

Interestingly, mice in the model group showed significant weight loss after CTX injection, likely attributable to drug-induced systemic toxicity. CTX is known to cause ovarian damage and disrupt sex hormone levels [7], which may influence eating behavior [50]. Additionally, it can induce intestinal inflammation and cachexia [51], collectively contributing to marked weight reduction. Although body weight partially recovered as inflammation subsided, ovarian reserve impairment and hormonal dysregulation persisted, likely restricting full weight restoration. Weight loss was also observed in FA-treated mice. However, FA administration helped preserve ovarian reserve, maintained near-normal levels of FSH, E_2_, and AMH, and conferred anti-inflammatory benefits [24]. These effects likely contributed to improved systemic conditions, as reflected by greater physical activity, attenuated weight loss, and a return to near-normal body weight by day 28 in FA-treated animals.

Notably, FA treatment significantly increased the ovarian expression of GDF9 and BMP15 in POI mice, with a particularly pronounced effect on GDF9. Both GDF9 [52] and BMP15 [53] jointly regulate follicle development and oocyte function. Mutations in these genes are associated with POI pathogenesis and impaired folliculogenesis [54,55]. Clinically, their reduced expression correlates with poor oocyte quality and unfavorable IVF outcomes [56]. In this study, the expression of both factors was significantly downregulated in POI model mice, while HFA treatment effectively restored their levels. These findings suggest that FA promotes follicle development and likely improves oocyte quality by upregulating these key folliculogenic growth factors.

ER serves as a central site for protein biosynthesis, folding, and post-translational modifications, and the maintenance of redox homeostasis within ER is essential for its normal function [57]. It has been established that OS, a key pathological contributor to POI [9,10], can disrupt ER redox equilibrium, promote protein misfolding, and thereby trigger ER stress. Conversely, persistent ER stress can further increase ROS generation through futile disulfide-bond cycling and mitochondrial calcium overload, establishing a detrimental “OS-ER stress” vicious cycle that collectively exacerbates ovarian damage [57,58]. In this study, ovarian tissues and KGN cells from the model group exhibited significantly elevated levels of ROS and MDA, accompanied by decreased T-SOD activity, indicating a severe disruption of the oxidative-antioxidative balance. As a natural phenolic antioxidant, FA effectively reduced ROS and MDA levels, enhanced T-SOD activity, and restored cellular redox homeostasis. Notably, ERO1α, a downstream target gene of ATF4, is transcriptionally induced during ER stress; however, its catalytic activity is coupled with H_2_O_2_ production [59], which may aggravate OS. Our results showed that FA treatment significantly decreased ERO1α protein expression in the ovaries of model mice, suggesting that FA may suppress the ATF4-ERO1α axis, thereby alleviating ER stress while concurrently blocking the secondary OS triggered by ERO1α activity. This coordinated action indicates that the protective effect of FA involves multi-level modulation of the OS-ER stress interplay.

Molecular docking and dynamics simulations revealed favorable binding properties of FA to both Grp78 and Perk proteins, providing a structural basis for the potential direct interaction between FA and ER stress-related proteins. Grp78 serves as a key regulatory protein and marker of the ER stress response. In the ovary, Grp78 expression fluctuates with physiological and pathological changes. A certain level of Grp78 supports primordial follicle activation, GC proliferation, follicle development, and embryogenesis [60,61,62], whereas Grp78 deficiency causes embryonic lethality [60]. Lin et al. [18] observed higher Grp78 levels in atretic goat follicles than healthy follicles, suggesting that severe ER stress may overwhelm adaptive UPR signaling and promote apoptosis, ultimately resulting in follicle atresia. In line with previous studies [63,64], our results showed that CTX treatment markedly upregulated Grp78 expression and induced ER stress in both mouse ovarian tissues and KGN cells. In contrast, FA administration significantly reduced Grp78 levels, reflecting a decreased burden of unfolded proteins within the ER. Consistent with the effect of the chemical chaperone 4-PBA, the downregulation of Grp78 following FA treatment likely indicates alleviation of the stress state, thereby corroborating the efficacy of FA in restoring ER homeostasis of POI models.

Furthermore, FA markedly inhibited the aberrant activation of the Perk/eIF2α/ATF4/CHOP pathway, showing a trend similar to that of 4-PBA. FA not only reduced the protein levels of p-Perk, p-eIF2α, ATF4, and CHOP but also downregulated the mRNA expression of *Perk* and *eIF2α*. Although Perk pathway activation primarily relies on post-translational modifications, the downregulation of relevant gene transcription further supports the sustained alleviation of ER stress. The introduction of the ER stress activator TM further validated that FA exerts its protective effects by regulating the ER stress pathway. Collectively, these results demonstrate that FA effectively intervenes in the excessive activation of ER stress signaling in POI models, blocking the transmission of downstream apoptotic signals.

ER stress-induced GCs apoptosis is a contributing factor to ovarian damage and follicle atresia [18,20,65]. The BCL-2 protein family, comprising anti-apoptotic members such as BCL-2 and BCL-xL and pro-apoptotic proteins like BAX, plays a central role in regulating apoptosis [66]. Activated Caspase 3 executes the final stages of cell death through cleavage of hundreds of key cellular substrates [67]. In this study, 4-OHCP exposure significantly compromised KGN cell viability and promoted apoptosis, reflected by elevated cleaved Caspase 3/Caspase 3 ratios, reduced levels of BCL-2 and BCL-xL proteins, and increased TUNEL-positive cells. Both LFA and HFA treatments effectively counteracted these changes. HFA markedly reduced apoptosis, and increased BCL-2 and BCL-xL expression. LFA also decreased apoptosis and increased BCL-xL, though its effect on BCL-2 was not statistically significant. Notably, total BAX protein levels remained unchanged across groups, likely because FA effectively blocked BAX activation without significantly altering its total expression. Moreover, FA exhibited anti-apoptotic efficacy comparable to the ER stress inhibitor 4-PBA. These results support that FA protects ovarian function in POI, at least in part, by suppressing ER stress-induced GC apoptosis.

DHEA is a pivotal precursor of androgens and estrogens. Since Casson et al. [68] first reported in 2000 that DHEA could enhance ovarian response in women with diminished ovarian reserve, it has been widely used as a supplementary treatment for women with POI undergoing assisted reproduction. In this study, DHEA was employed as a positive control. The results confirmed that DHEA alleviates follicle loss and hormonal imbalances, while also demonstrating antioxidant activity. Notably, DHEA was observed in this study to modulate ATF4, potentially influencing the redox homeostasis of ovarian cells through the ATF4-ERO1α axis. However, DHEA did not show significant regulatory effects on key molecules of the Perk pathway, including p-Perk and CHOP, at either the protein or gene level. This suggests that the protective effects of DHEA in POI may be mediated through other intricate cellular processes.

However, the present has several limitations. First, ovarian function was assessed by measuring follicle development-related factors, without evaluating follicle maturation, ovulation, or subsequent pregnancy outcomes. Second, as the experiments were conducted in a mouse model, the clinical relevance remains limited, and the mechanisms identified may not fully translate to humans. Future studies should further investigate FA’s regulatory effects on key ER stress proteins and its potential interactions with other signaling pathways. In addition, research on the influence of FA on long-term reproductive outcomes is warranted to facilitate its clinical translation.

In summary, this study systematically elucidates the protective effects of FA on a CTX-induced POI model and its underlying mechanisms. The results demonstrate that FA not only significantly improves ovarian morphology and functional indices, restores sex hormone balance, and promotes follicle development but also effectively alleviates OS, suppresses Grp78 expression, and inhibits the aberrant activation of the key ER stress pathway Perk/eIF2α/ATF4/CHOP, thereby reducing granulosa cell apoptosis. Notably, by downregulating ERO1α expression, FA effectively suppresses the increase in cellular oxidative pressure and mitigates oxidative damage, suggesting that FA may exert a synergistic protective effect through the simultaneous regulation of OS and ER stress. This study provides experimental evidence supporting FA as a potential therapeutic candidate for POI and lays a theoretical foundation for the further development of ovarian protection strategies based on the modulation of ER homeostasis.

## 5. Conclusions

In conclusion, the present study reveals that FA significantly alleviates CTX-induced POI in mice. FA treatment preserves ovarian reserve, restores hormone balance, enhances folliculogenesis, and attenuates OS. Mechanistically, FA suppresses Grp78 levels and inhibits the overactivation of the Perk/eIF2α/ATF4/CHOP signaling pathway, thereby attenuating ER stress and suppressing GC apoptosis. Additionally, FA decreases ERO1α expression, interrupting the OS-ER stress vicious cycle. Collectively, these results identify FA as a promising natural agent for ameliorating ovarian dysfunction in POI, chiefly through the coordinated regulation of oxidative and ER stress pathways. The present work offers substantive experimental support for the potential therapeutic use of FA in POI treatment.

## Figures and Tables

**Table 1:** The primers for real-time qPCR (mouse).

Gene	Forward Primer (5′-3′)	Reverse Primer (3′-5′)
* β-actin *	CACTGTCGAGTCGCGTCC	TCATCCATGGCGAACTGGTG
* Amh *	TACTCGGGACACCCGCTATT	CTCAGGGTGGCACCTTCTCT
*Grp78*	CGTGTGTGTGAGACCAGAAC	CAGTCAGGCAGGAGTCTTAGG
*Perk*	CAGTGGGATTTGGACGTGGG	GAAGTTTTGTGGGTGCCCTCTG
*eIF2α*	CACATCCACTTCAGAATGCCG	CATAGGCCCCCATTTCAGCA
*Atf4*	CCTATAAAGGCTTGCGGCCA	GATTTCGTGAAGAGCGCCAT
*Chop*	CCCCAGGAAACGAAGAGGAAG	ATGTGCGTGTGACCTCTGTT

**Table 2:** The primers for Real-time qPCR (human).

Gene	Forward Primer (5′-3′)	Reverse Primer (3′-5′)
* β-actin *	CATGTACGTTGCTATCCAGGC	CTCCTTAATGTCACGCACGAT
*Grp78*	CATCACGCCGTCCTATGTCG	CGTCAAAGACCGTGTTCTCG
*Perk*	ACGATGAGACAGAGTTGCGAC	ATCCAAGGCAGCAATTCTCCC
*eIF2α*	TGGTGAATGTCAGATCCATTGC	TAGAACGGATACGCCTTCTGG
*Atf4*	ATGACCGAAATGAGCTTCCTG	GCTGGAGAACCCATGAGGT
*Chop*	GGAAACAGAGTGGTCATTCCC	CTGCTTGAGCCGTTCATTCTC

## Data Availability

Data is contained within the article or Supplementary Materials.

## Supplementary Materials

The following supporting information can be downloaded at: https://www.mdpi.com/article/10.3390/biomedicines14030714/s1, Figure S1: Representative images of the morphology of the ovaries and uterus in each group; Table S1: Model vs. Control gene_all; Table S2: FA vs. Model gene_all; Table S3: FA vs. Model KEGG enrichment_top10; Table S4: FA vs. Model GO_DOWN; Table S5: FA vs. Model GO_UP.

## Abbreviations

The following abbreviations are used in this manuscript:

4-OHCP	4-hydroperoxy Cyclophosphamide
4-PBA	4-Phenylbutyric Acid
AMH	anti-Müllerian Hormone
ATF4	Activating Transcription Factor 4
BAX	BCL2-associated X protein
BCL-2	B-cell lymphoma-2
BMP15	Bone Morphogenetic Protein 15
CHOP	C/EBP-Homologous Protein
CMC-Na	Sodium Carboxymethyl Cellulose
CTX	Cyclophosphamide
DHE	Dihydroethidium
DHEA	Dehydroepiandrosterone
DMSO	Dimethyl Sulfoxide
E_2_	Estradiol
eIF2α	Eukaryotic Translation Initiation Factor 2α
FA	Ferulic Acid
FBS	Fetal Bovine Serum
FSH	Follicle-Stimulating Hormone
GCs	Granulosa Cells
GDF9	Growth Differentiation Factor 9
Grp78	Glucose-Regulated Protein-78
HE	Hematoxylin-Eosin
HRT	Hormone Replacement Therapy
HSP70	Heat Shock Protein 70
IHC	Immunohistochemistry
IVF	in vitro Fertilization
MDA	Malondialdehyde
OS	Oxidative Stress
Perk	Protein Kinase R-like ER Kinase
POI	Premature Ovarian Insufficiency
ROS	Reactive Oxygen Species
RT-qPCR	Real-time Quantitative Polymerase Chain Reaction
SOD	Superoxide Dismutase
TGF-β	Transforming Growth Factor-β
TM	Tunicamycin
UPR	Unfolded Protein Response
WB	Western Blotting
