# Supplementary figures and images for: Ferulic Acid Alleviates Chemotherapy-Induced POI by Targeting the Grp78 and Perk-eIF2α-ATF4-CHOP Pathway to Attenuate Endoplasmic Reticulum Stress
Source: Biomedicines. 2026 Mar 19;14(3):714. doi: 10.3390/biomedicines14030714 (PMC13024552; doi:10.3390/biomedicines14030714)

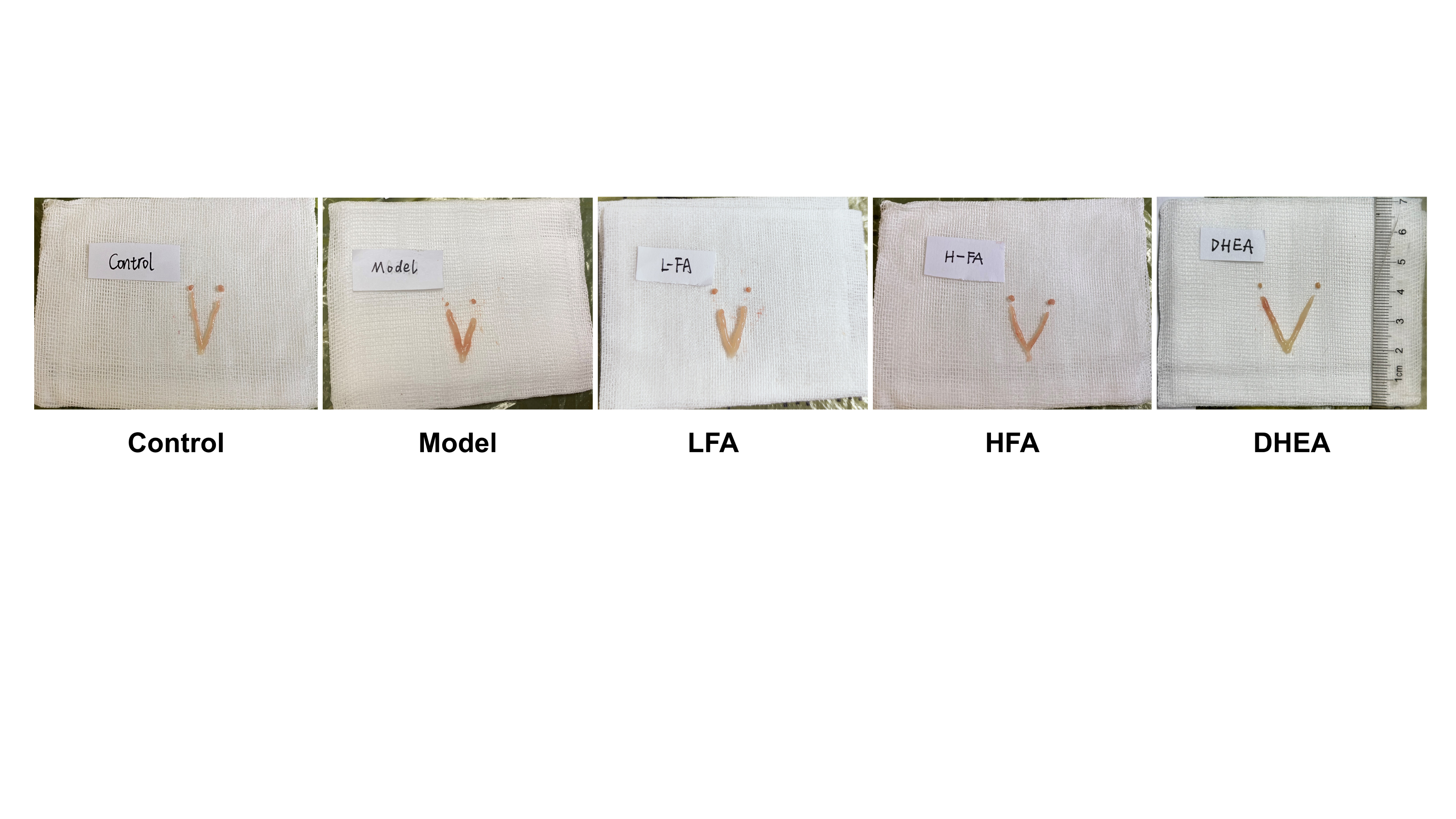

Supplement: Supplementary file 1 [file biomedicines-14-00714-s001.zip › Supplymentary Figure S1.tif]
